# Phenotypic Characterization of Transgenic Mice Overexpressing Neuregulin-1

**DOI:** 10.1371/journal.pone.0014185

**Published:** 2010-12-09

**Authors:** Taisuke Kato, Atsushi Kasai, Makoto Mizuno, Liang Fengyi, Norihito Shintani, Sadaaki Maeda, Minesuke Yokoyama, Miwako Ozaki, Hiroyuki Nawa

**Affiliations:** 1 Department of Molecular Neurobiology, Brain Research Institute, Niigata University, Niigata, Japan; 2 Department of Pharmacotherapeutics, Faculty of Pharmaceutical Sciences, Setsunan University, Osaka, Japan; 3 Center for Transdisciplinary Research, Niigata University, Niigata, Japan; 4 Department of Anatomy, Yong Loo Lin School of Medicine, National University of Singapore, Singapore, Singapore; 5 Department of Molecular Neuropharmacology, Graduate School of Pharmaceutical Sciences, Osaka University, Suita, Osaka, Japan; 6 Center for Bioresource-Based Researches, Brain Research Institute, Niigata University, Niigata, Japan; 7 Consolidated Research Institute for Advanced Science and Medical Care, Waseda University, Tokyo, Japan; 8 Neuroscience and Neuroengineering Group, Waseda Bioscience Institute of Singapore (WABIOS), Helios, Singapore; University of Queensland, Australia

## Abstract

**Background:**

Neuregulin-1 (NRG1) is one of the susceptibility genes for schizophrenia and implicated in the neurotrophic regulation of GABAergic and dopaminergic neurons, myelination, and NMDA receptor function. Postmortem studies often indicate a pathologic association of increased NRG1 expression or signaling with this illness. However, the psychobehavioral implication of NRG1 signaling has mainly been investigated using hypomorphic mutant mice for individual NRG1 splice variants.

**Methodology/Principal Findings:**

To assess the behavioral impact of hyper NRG1 signaling, we generated and analyzed two independent mouse transgenic (Tg) lines carrying the transgene of green fluorescent protein (GFP)-tagged type-1 NRG1 cDNA. The promoter of elongation-factor 1α gene drove ubiquitous expression of GFP-tagged NRG1 in the whole brain. As compared to control littermates, both heterozygous NRG1-Tg lines showed increased locomotor activity, a nonsignificant trend toward decreasing prepulse inhibition, and decreased context-dependent fear learning but exhibited normal levels of tone-dependent learning. In addition, social interaction scores in both Tg lines were reduced in an isolation-induced resident-intruder test. There were also phenotypic increases in a GABAergic marker (parvalbumin) as well as in myelination markers (myelin basic protein and 2′,3′-cyclic nucleotide 3′-phosphodiesterase) in their frontal cortex, indicating the authenticity of NRG1 hyper-signaling, although there were marked decreases in tyrosine hydroxylase levels and dopamine content in the hippocampus.

**Conclusions:**

These findings suggest that aberrant hyper-signals of NRG1 also disrupt various cognitive and behavioral processes. Thus, neuropathological implication of hyper NRG1 signaling in psychiatric diseases should be evaluated with further experimentation.

## Introduction

A genetic association between the neuregulin-1 (NRG1) gene and schizophrenia has been documented in various human populations. However, the exact biological relationship is still unclear [1]–[3]. Many model studies have used NRG1 hypomorphic mutant mice to study the phenotypic consequences of decreased NRG1 signaling as well as its pathologic contribution to schizophrenia [2], [4]–[13]. Differential promoter usage and alternative splicing produce a large variety of structural variants of NRG1 precursor proteins. For example, the type-1, -2, and -4 subgroups of NRG1 contain a immunoglobulin-like domain and a transmembrane domain while the type-3 variant carries two transmembrane domains and a cystein-rich domain [14], [15]. Mutant mice deficient in NRG1 have been found to exhibit schizophrenia-associated behavioral abnormalities in sensorimotor gating [2], [4], social interactions [5], [9]–[11], latent inhibition [12], and locomotor activity [8], [13], although neurobehavioral features of the individual mutants significantly differ depending upon the targeted isoforms of NRG1 [2], [6], [7]–[13]. NRG1 has neurotrophic activities to promote NMDA receptor expression, GABA synthesis, and myelination, all of which are diminished in postmortem brain of schizophrenia patients [14], [15]. The animal and patient studies suggest that decreased NRG1 signals are responsible for the pathophysiology of schizophrenia [14], [15]. However, this argument is not supported by all types of studies. For example, postmortem studies report that higher levels of type-1 and type-4 NRG1 mRNA are present in the hippocampus and prefrontal cortex of schizophrenic patients as well as in patients' lymphocytes, compared to control subjects [16]–[19]. Similarly, the up-regulation of the NRG1 protein or its signaling is detected in the brains of schizophrenic patients [20], [21]. Thus, these patient studies rather suggest a biological link between increased NRG1 signaling and the pathophysiology of schizophrenia. As the type-1 NRG1 variant display marked mRNA increase in patients' postmortem brain and single nucleotide polymorphisms (SNPs) of its corresponding genome locus are implicated in genetic vulnerability to schizophrenia [18], [19], we have established mouse transgenic lines carrying the transgene of mouse type-1 NRG1 cDNA and examined whether NRG1 hypermorphic mice, as opposed to NRG1 hypomorphic mice, are an appropriate animal model for schizophrenia. In the present investigation, we analyzed two independent transgenic (Tg) mouse lines to minimize the effects of the transgene insertion in their host genome as well as those of the genetic inhomogeneity of mouse genetic background. Further, we examined neurochemical consequences of NRG1 overexpression in several neuronal and glial markers in one of the Tg lines. Behavioral similarity and difference between the present Tg mice and reported NRG1-knockout mice are also discussed.

## Results

### Generation of transgenic mice overexpressing NRG1

**Figure 1:**
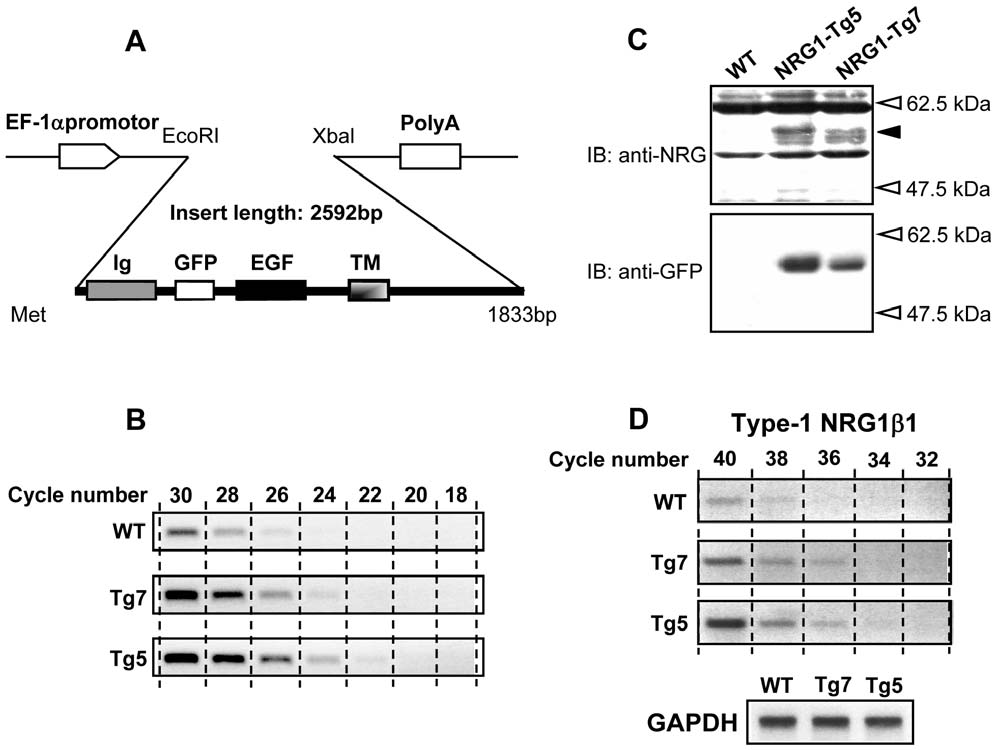
Establishment of GFP-tagged NRG1 transgenic mice with EF1α-promoter. (**A**) A schematic illustration of a transgene construct carrying EF-1α genomic promoter, NRG1β1 cDNA, GFP-tag insertion, and poly A signal. (**B**) Estimation of the copy number of the transgene by PCR. The exon 3 fragment of NRG1 genome was amplified with 18–30 cycles using tail DNA from Tg5 and Tg7 and separated in an agarose-gel. (**C**) Protein lysate was prepared from whole brain of adult male NRG1-Tg mice (Tg5 and Tg7) and WT littermate and subjected to immunoblotting with anti-NRG1 and anti-GFP antibodies. A closed arrowhead marks the transgene products. (**D**) Quantification of mRNA levels for type-1 NRG1 by RT-PCR. cDNA fragments specific for type-1 NRG1 and GAPDH mRNAs were amplified in the presence of SYBR Green I. PCR amplification curves and difference in Ct were analyzed by a real-time temperature cycler (LightCycler, Roche Molecular Biochemicals). For figure display, RT-PCR products were also separated by agarose-electrophoresis and visualized with ethidium bromide staining.

We constructed the Tg vector that contained the promoter of a house keeping gene, elongation-factor 1α (EF1α) and GFP-tagged NRG1β1 cDNA. The GFP-tag facilitated transgene expression and detection in mice. The Tg vector was injected to fertilized eggs to generate transgenic mice (**Fig. 1A**). The modification of GFP tagging is known not to affect NRG1 function [22]. We selected two independent NRG1-Tg lines, Tg5 and Tg7, which were viable and healthy with normal body weights and reproduction (data not shown). The number of transgene copies integrated in genome was estimated by polymerase chain reaction (PCR). Densitometric measurement revealed that PCR products from Tg5 DNA first appeared at ∼22 cycle and that from Tg7 at ∼24 cycle, 3–4 and 1–2 cycle earlier than the emergence of wild type (WT) mice product (2 copies of wild allele) respectively (**Fig. 1B**). Based on the present efficiency of PCR amplification (1.6±0.1 fold/cycle), we estimated that Tg5 mouse contained 5∼6 copies of the transgene and Tg7 mouse carried ∼2 copies. To confirm the expression of the transgene in the brain, we performed an immunoblotting analysis with anti-NRG1 and anti-GFP antibodies. There were NRG1-like and GFP-like immunoreactivities at the same size (55 kDa) in whole brain lysates of Tg5 and Tg7 (**Fig. 1C**). The size approximately matches the sum of molecular weights of GFP (25 kDa) and a shedded mature form of NRG1β1 (30 kDa). The Tg5 line contained higher levels of the transgene product than the Tg7 line. To confirm the overexpression of type-1 NRG1 mRNA in the transgenic mice, we carried out real-time quantitative reverse transcription (RT)-PCR for type-1 NRG1 mRNA in the presence of SYBR green I. Calculation of PCR amplification curves and the threshold cycle (Ct) suggested that the transgenic mice expressed approximately 4.3-fold (Tg5) and 2.2-fold (Tg7) higher levels of type-1 NRG1 mRNA than wild type littermates. The mRNA increases were also apparent in agarose gel electrophoresis (**Fig. 1D**).

**Figure 2:**
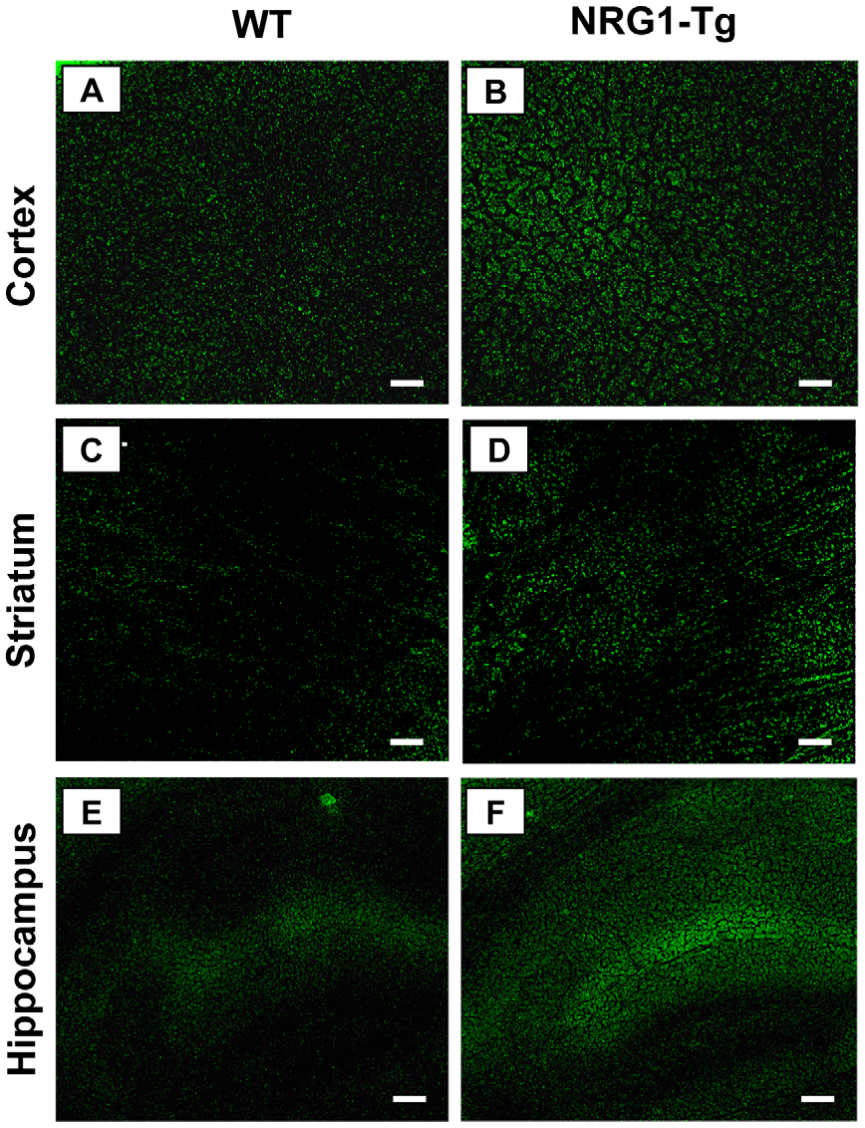
Detection of the transgene expression by GFP fluorescence in the brain. The transgenic line (NRG1-Tg5) was fixed and slices were prepared from their brain. The green fluorescence by GFP protein was examined in the cortex (**A, B**), striatum (**C, D**), and hippocampus (**E, F**), and compared between male NRG1-Tg and WT mice. Scale bars = 50 µm in A, B, and 100 µm in C–F. Note: Fixed brain of WT mice also exhibits autofluorescence but its intensity is lower than that of NRG1-Tg mice.

The expression pattern of the GFP-NRG1 protein within the Tg5 line was examined by GFP-fluorescence. Under the control of the EF1α promoter, GFP signals were ubiquitously and homogeneously distributed throughout the brain including the cerebral cortex, striatum, and hippocampus (**Fig. 2A–F**). There was a similar distribution pattern of GFP signals in the Tg7 line (data not shown).

### Gross physical conditions of NRG1-Tg mice

The physical abilities of mice, such as sensory ability, motor reflex and coordination, directly and indirectly influence performance scores in behavioral tests. To estimate health and physical conditions of NRG1-Tg mice, we investigated various physical and behavioral parameters and compared between genotypes (transgenic vs wild), between lines (Tg5 vs Tg7) and between genders. MANOVA revealed that there was no significant main effect of genotype [F(16, 30)  = 1.249, *P* = 0.290] or any significant interactions of genotype with line [F(16, 39)  = 0.974, *P* = 0.506] or gender [F(16, 39)  = 118.50, *P* = 0.226]. This suggests that the NRG1 transgene did not significantly influence gross health and physical conditions in NRG1-Tg mice. As MANOVA also detected significant main effects and/or interactions of gender and Tg line (see details in **Table S1 and Table S2**), we analyzed two Tg lines independently and tested a gender x genotype interaction in the following individual behavioral tests.

### Hyperlocomotor activity of NRG1-Tg mice in a novel environment

**Figure 3:**
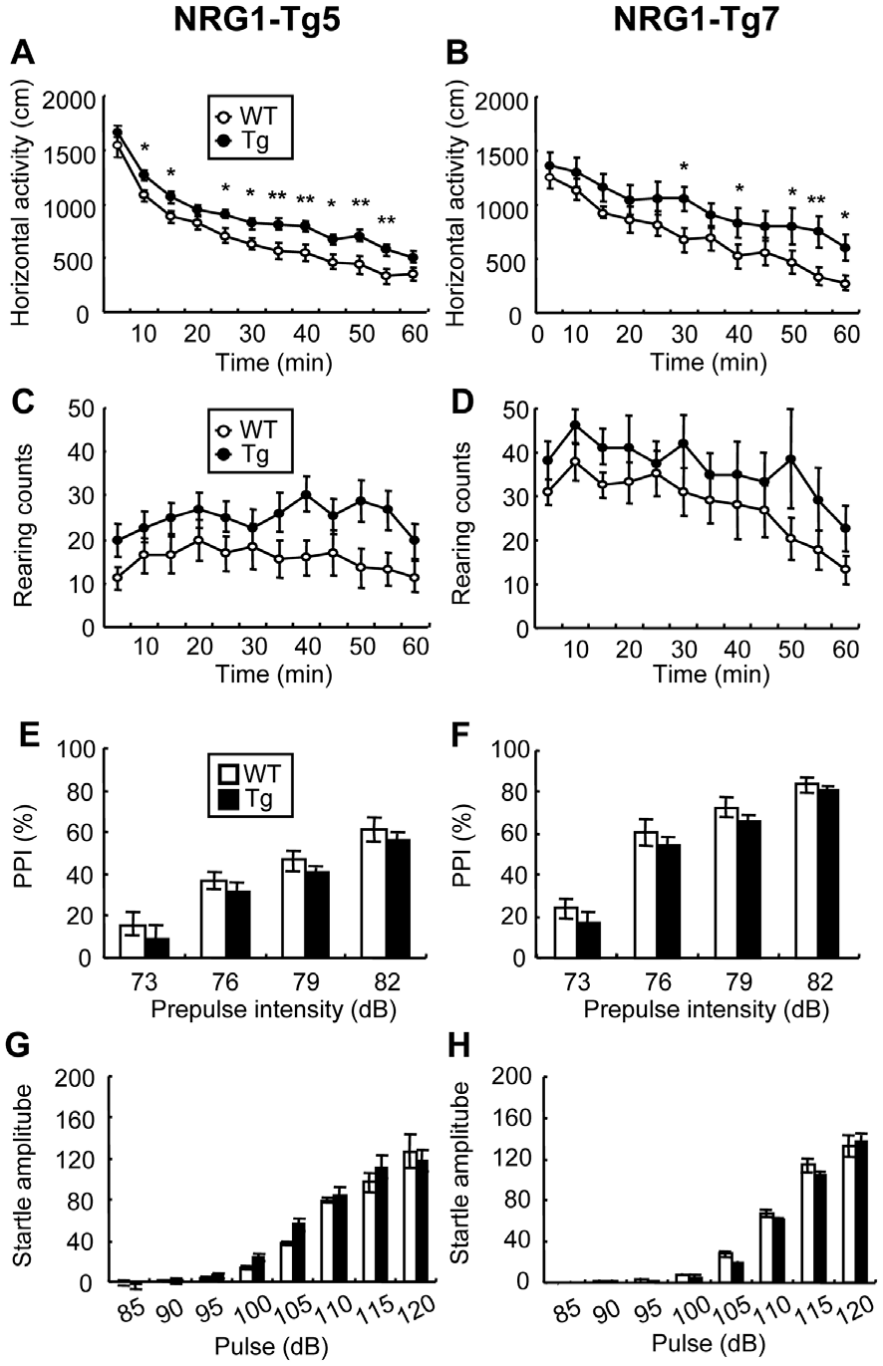
Locomotor activity and sensorimotor gating of NRG1-Tg mice. Behavioral traits were compared between NRG1-Tg5 mice and WT littermates (**A, C, E, G**) and between NRG1-Tg7 mice and WT littermates (**B, D, F, H**) at the adult stage (postnatal day PND 56–84). (**A, B**) Horizontal locomotor activity was scored every 5 min in a novel environment. (**C, D**) Rearing behavior was counted similarly [N = 23 (male: N = 9, female: N = 14) for Tg5, N = 18 (male: N = 7, female: N = 11) for WT; and N = 15 (male: N = 8, female: N = 7) for Tg7, N = 14 (male: N = 6, female: N = 8) for WT]. There was neither significant or marginal tread in a gender x genotype interaction; Tg5: F(1, 37)  = 1.06 (locomotor activity) and 0.70 (rearing behavior), *P* = 0.31 (locomotor activity) and 0.79 (rearing behavior); Tg7: F(1, 25)  = 0.01 (locomotor activity) and 0.11 (rearing behavior), *P* = 0.94 (locomotor activity) and 0.69 (rearing behavior)]. (**E, F**) Prepulse inhibition (PPI) percentages are shown with prepulses of 73, 76, 79 and 82 dB [N = 23 (male: N = 9, female: N = 14) for Tg5, N = 18 (male: N = 7, female: N = 11) for WT; and N = 15 (male: N = 8, female: N = 7) for Tg7, N = 14 (male: N = 6, female: N = 8) for WT]. There was neither significant or marginal tread in a gender x genotype interaction; Tg5: F(1, 37)  = 1.90, *P* = 0.18; Tg7: F(1, 19)  = 0.11, *P* = 0.74]. (**G, H**) Relative amplitudes of startle responses to white noise at 75, 80, 85, 90, 95, 100, 105, 110, 115 and 120 dB tones are shown [N = 12 (male: N = 6, female: N = 6) for Tg 5, N = 10 [male: N = 5, female: N = 5) for WT; and N = 10 (male: N = 5, female: N = 5) for Tg7, N = 10 (male: N = 5, female: N = 5) for WT]. There was neither significant or marginal tread in a gender x genotype interaction; Tg5: F(1, 18)  = 0.64, *P* = 0.43; Tg7: F(1, 15)  = 1.59, *P* = 0.23]. Data are expressed as mean±S.E.M. *P<0.05, **P<0.01 compared to WT mice by Fisher's LSD.

We assessed behavioral pathology of adult NRG1-Tg mice by measuring locomotor activity, prepulse inhibition (PPI), fear learning, and social interaction, which are often implicated in schizophrenia animal models. First, we used an open field task to examine the locomotor behavior of the NRG1 Tg lines, Tg5 and Tg7. A two-way repeated ANOVA using a between-subjects factor of genotype and a within-subjects factor of time revealed a significant main effect of genotype in both lines [Tg5: F(1, 40)  = 10.79, *P*<0.05; Tg7: F(1, 27)  = 5.19, *P*<0.05]. These results indicated that increases in NRG1 expression led to hyperactivity in a novel environment (**Fig. 3A, B**). The significant effect of time [Tg5: F(11, 440)  = 118.50, *P*<0.001; Tg7: F(11, 297)  = 25.03; *P*<0.001] and the lack of interaction between genotype and time [Tg5: F(11, 440)  = 0.60, *P* = 0.83; Tg7: F(11, 297)  = 0.72, *P* = 0.72] suggested that mice exhibited a decrease in locomotor activity over time. Furthermore, the rate of habituation was not significantly different between NRG1-Tg and WT mice. The novelty-induced rearing behavior of NRG1-Tg mice was simultaneously scored and compared with that of wild littermates. There were no significant differences in rearing behavior [genotype, Tg5: F(1, 40)  = 3.47, *P* = 0.07; Tg7: F(1, 27)  = 2.17, *P* = 0.15] (**Fig. 3C, D**).

### NRG1-Tg mice exhibit normal prepulse inhibition and startle responses

Using different prepulse intensities, we examined and compared PPI levels of adult NRG1-Tg mice and WT littermates from the two Tg lines. Both NRG1-Tg mice revealed a non-significant trend toward decreasing PPI levels compared to WT mice [genotype, Tg5: F(1, 39)  = 3.39, *P* = 0.073; Tg7: F(1, 21)  = 3.36, *P* = 0.081]. PPI levels were dependent on prepulse intensity [Tg5: F(3, 117)  = 64.54, *P*<0.001; Tg7: F(3, 63)  = 76.9, *P*<0.001]. There was no significant interaction between genotype and prepulse intensity [Tg5: F(3, 117)  = 0.02, *P* = 0.99; Tg7: F(3, 63)  = 0.05, *P* = 0.99] (**Fig. 3E, F**). In pulse-alone startle responses, the amplitude of startle responses was dependent on pulse intensity [Tg5: F(7, 140)  = 90.14, *P*<0.001; Tg7: F(7, 119)  = 73.61, *P*<0.001]. There was no difference between the genotypes in both lines [genotype, Tg5: F(1, 20)  = 0.21, *P* = 0.65; Tg7: F(1, 17)  = 0.06, *P* = 0.82] and no significant interaction between genotype and tone intensity [Tg5: F(7, 140)  = 0.76, *P* = 0.62; Tg7: F(7, 119)  = 1.15, *P* = 0.19] (**Fig. 3G, H**).

### Impaired context-dependent fear learning in NRG1-Tg mice

**Figure 4:**
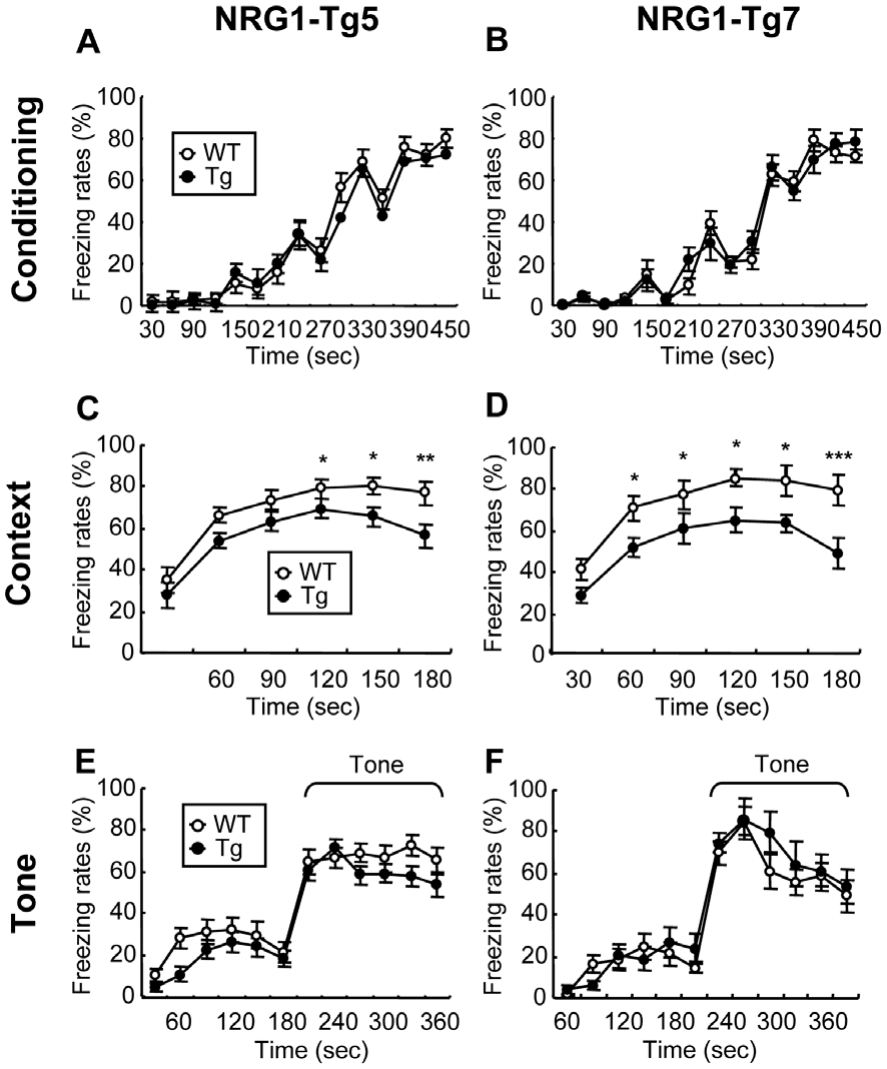
Context-dependent and tone-dependent fear learning in NRG1-Tg mice. Learning ability was compared between NRG1-Tg5 mice and WT littermates (**A, C, E**) and between NRG1-Tg7 mice and WT littermates (**B, D, F**). NRG1-Tg mice and WT littermates were subjected to shock-paired contextual conditioning with a tone cue. One day after conditioning, their learning performance was measured in the presence of a contextual or tone cue. (**A, B**) Freezing rates (time %) were compared between NRG1-Tg mice and WT littermates during conditioning. (**C, D**) Freezing rates during context exposure are shown. (**E, F**) Freezing rates were compared between NRG1-Tg mice and WT littermates during tone exposure [N = 21 (male: N = 11, female: N = 10) for Tg5, N = 18 (male: N = 10, female: N = 8) for WT; and N = 12 (male: N = 6, female: N = 6) for Tg7, N = 11 (male: N = 6, female: N = 5) for WT]. There was neither significant or marginal tread in a gender × genotype interaction; Tg5: F(1, 35)  = 0.06 (conditioning), 0.01 (context) and 0.19 (tone), *P* = 0.81 (conditioning), 0.99 (context) and 0.66 (tone); Tg7: F(1, 19)  = 0.01 (conditioning), 0.10 (context) and 0.91 (tone), *P* = 0.94 (conditioning), 0.76 (context) and 0.35 (tone). Data are expressed as mean±S.E.M. *P<0.05, **P<0.01, *** <0.001 compared to WT mice by Fisher's LSD.

The effect of NRG1 overexpression on learning performance was examined in adulthood by measuring freezing behavior following fear conditioning. In this task, electric shock was coupled with a context plus a tone. In both mouse Tg lines, there was no significant difference in freezing rates during conditioning between NRG1-Tg mice and their WT littermates [genotype, Tg5: F(1, 37)  = 0.68, *P* = 0.42; Tg7: F(1, 21)  = 0.05, *P* = 0.83] (**Fig. 4A, B**). This finding suggests that there was no significant influence of the NRG1 transgene on shock sensitivities. A two-way repeated ANOVA using a between subjects factor of genotype and a within-subjects factor of time detected a significant effect of genotype on freezing rates when the test was coupled with the context [Tg5: F(1, 37)  = 8.45, *P*<0.01; Tg7: F(1, 21)  = 15.86, *P*<0.01]. However, there was no interaction between genotype and time [Tg5: F(5, 185)  = 0.66, *P* = 0.65; Tg7: F(5, 105)  = 0.66, *P* = 0.65] (**Fig. 4C, D**). In contrast, there was no significant difference in tone-dependent learning between NRG1-Tg mice and their WT littermates [genotype: Tg5: F(1, 37)  = 1.84, *P* = 0.18; Tg7: F(1, 21)  = 0.70, *P* = 0.41] (**Fig. 4E, F**). These results indicate that the overexpression of NRG1 in the Tg mice specifically impairs context-dependent learning ability.

### Social behavior of NRG1-Tg mice in an isolation-induced resident-intruder test

**Figure 5:**
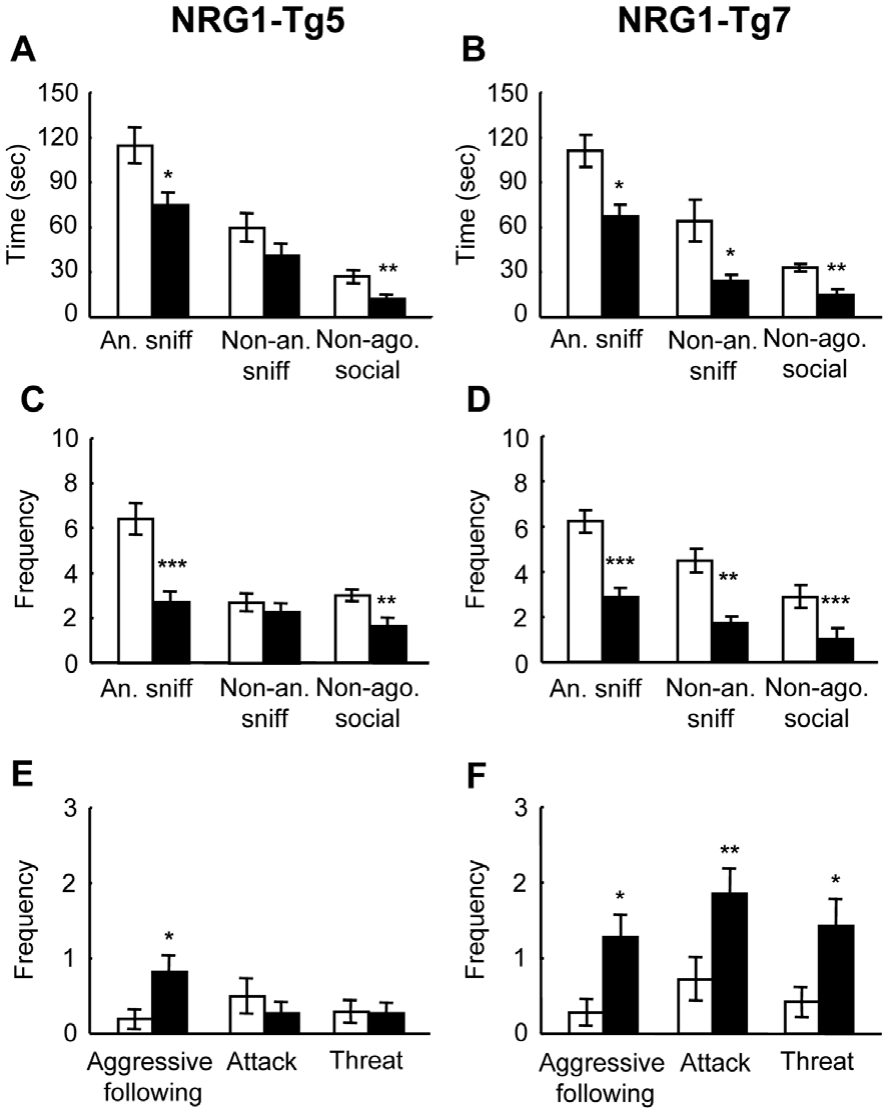
Isolation-induced resident-intruder test. NRG1-Tg5 mice (**A, C, E**) and Tg7 mice (**B, D, F**) and their WT littermates were subjected to a isolation-induced resident-intruder test. (**A–D**) Social scores of anogenital sniffing (An. sniff), non-anogenital sniffing (Non-an. sniff) and non-agonistic social behaviors (Non-ago. social) were measured over a 10-min period. The non-agonistic behaviors represent grooming and lying down next to each other of resident mice. (**E, F**) Aggressive behaviors, which represent aggressive following, attacks and threats, were counted in parallel. (**A, B**) Time spent by the resident males actively pursuing social behaviors. (**C, D**) The frequency of social behaviors in the resident males is shown. (**E, F**) The frequency of aggressive behaviors of the resident males was compared between the NRG1-Tg mice and their WT littermates (N = 11 for Tg5, N = 10 for WT; and N = 7 for Tg7, N = 7 for WT, all males). Data are expressed as mean±S.E.M. *P<0.05, **P<0.01, ***P<0.001 compared to WT littermates by unpaired two-tailed t-test.

The effect of the NRG1 transgene on social behavior was examined using an isolation-induced resident-intruder test. In this assay, a resident male, who was previously housed alone, was exposed to an unfamiliar intruder male. The social (**Fig. 5A–D**) and aggressive behaviors (**Fig. 5E, F**) of the resident male in response to the intruder were monitored and scored. The NRG1-Tg residents displayed a significant decrease in the duration of social behaviors compared to those of WT male residents (anogenital sniffing, Tg5: *P*<0.05; Tg7: *P*<0.05; non-anogenital sniffing, Tg7: *P*<0.05, non-agonistic social behavior, Tg5: *P*<0.01; Tg7: *P*<0.01, unpaired two tailed t-test) (**Fig. 5A, B**). There was no significant difference in non-anogenital sniffing between Tg5 and wild mice, however (non-anogenital sniffing, Tg5: *P* = 0.13, unpaired two tailed t-test). This trend was also supported by a decrease in the frequency of these behaviors (anogenital sniffing, Tg5: *P*<0.001; Tg7: *P*<0.001; non-anogenital sniffing, Tg5: *P* = 0.42; Tg7: *P*<0.05; non-agonistic social behavior, Tg5: *P*<0.01; Tg7: *P*<0.01, unpaired two tailed t-test) (**Fig. 5C, D**). In aggressive behaviors, Tg5 male residents displayed an increase in frequency of aggressive following behavior (*P*<0.05), but the frequency of attack and threat behaviors of Tg5 residents was indistinguishable from that of WT resident males (attack: *P* = 0.39; threat: *P* = 0.90) (**Fig. 5E**). In contrast, Tg7 resident males showed an increase in all indices of aggressive behaviors (aggressive following: *P*<0.05 attack: *P*<0.01; threat: *P*<0.05) (**Fig. 5F**). Although the Tg line-specific behavioral changes require further investigation, our results from the resident-intruder test indicate that NRG1 overexpression has significant influences on social behavior.

### Analysis of neurochemical markers for excitatory and inhibitory neurons and glial cells

**Figure 6:**
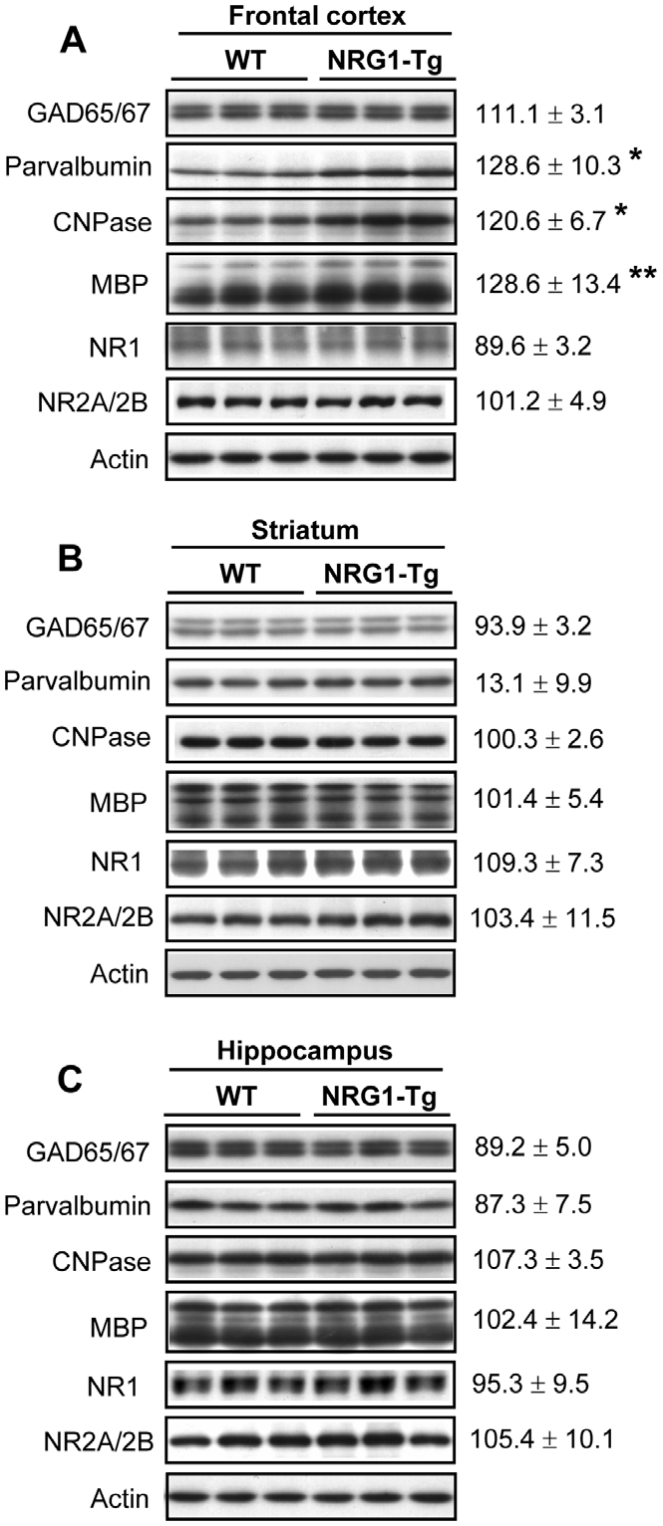
The expression of GABA-, myelin-, and excitatory synapse-associated molecular markers. Protein extract was prepared from (**A**) frontal cortex, (**B**) striatum, and (**C**) hippocampus and subjected to immunoblotting with antibodies directed against the GABAergic markers (GAD65/67 and parvalbumin), oligodendrocyte markers (CNPase and MBP), and excitatory synaptic markers (NR1 and NR2A/2B). Immunoreactivity on immunoblots was measured by densitometric analysis, and normalized to β-actin levels. Percentage ratio to that of WT littermates was calculated (mean±S.E.M, N = 5, all males) and analyzed by unpaired two-tailed t-test.

NRG1 is involved in the regulation of GABAergic development, myelin formation, and NMDA receptor expression and function [23]–[26]. To explore whether the NRG1 transgene influenced these processes, we determined protein levels of molecular markers for GABAergic neurons, oligodendrocytes, and excitatory synapses and compared those between Tg5 mice and their WT littermates. Immunoblotting revealed that the immunoreactivity for parvalbumin, one of the phenotypic markers for cortical GABAergic neurons, was elevated in the frontal cortex of Tg5 mice (*P*<0.05) (**Fig. 6A**). Furthermore, we also found significant increases in myelin-basic protein (MBP, *P*<0.01) and 2′,3′-cyclic nucleotide 3′-phosphodiesterase (CNPase, *P*<0.05) in the same region (**Fig. 6A**). This result at least verified the hyper-signaling of NRG1 expressed from the transgene. There were no significant alterations in protein levels of glutamate decarboxylase (GAD) 65/67, NMDA receptor1 (NR1), and NMDA receptor 2A/2B (NR2A/2B) in the fontal cortex (**Fig. 6A**) as well as in all markers examined in other brain regions (**Fig. 6B, C**).

### Analysis of dopaminergic markers, tissue contents of dopamine and its metabolites in NRG1-Tg mice

**Figure 7:**
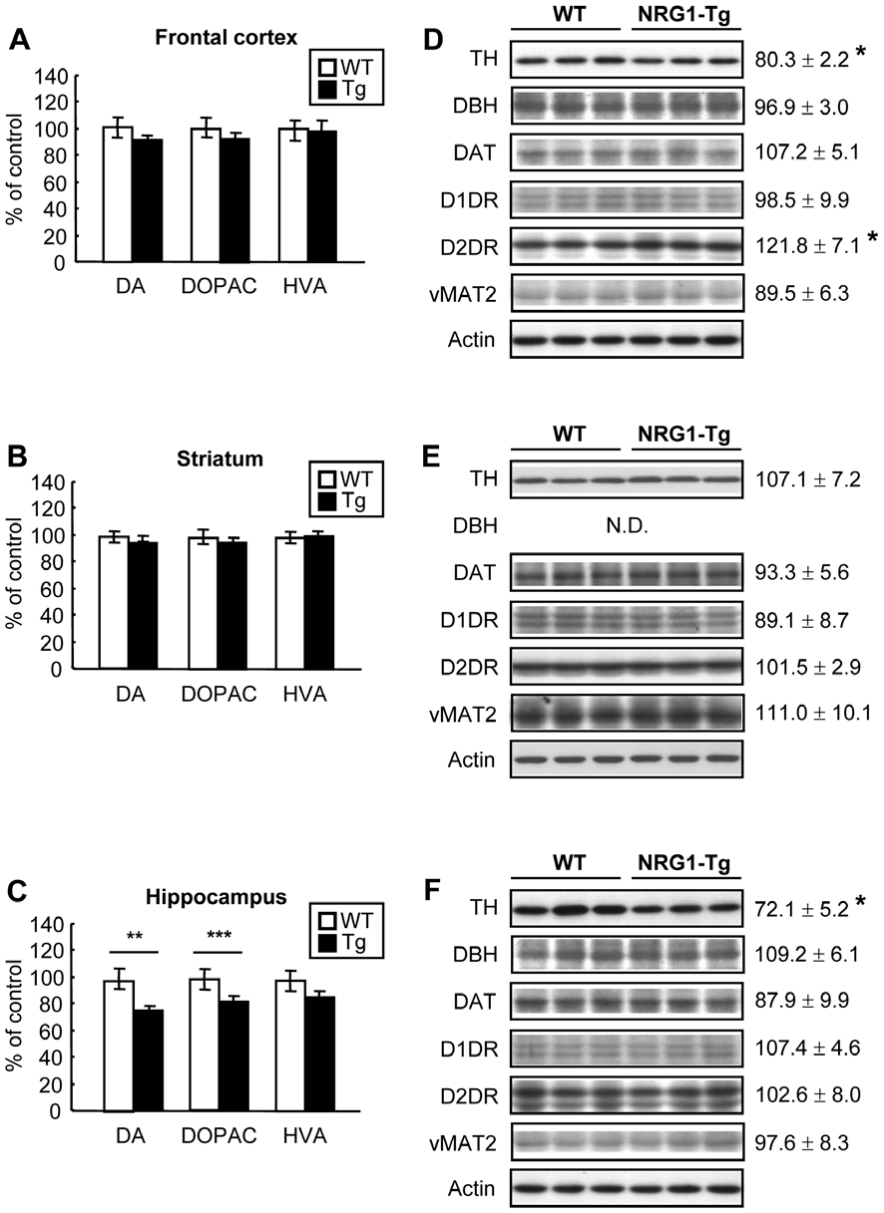
Analysis of dopamine metabolism and neurochemical markers for dopaminergic neurons of NRG1 transgenic mice. Levels of dopamine and its metabolites (DOPAC and HVA) (**A, B, C**) as well as those of dopamine-related molecular markers (**D, E, F**) were measured in (**A, D**) frontal cortex, (**B, E**) striatum and (**C, F**) hippocampus of NRG1-Tg5 and WT mice at the adult stage. Typical immunoblots for TH, DBH, DAT, D1DR, D2DR, and vMAT2 were displayed. Each immunoreactivity was measured by densitometric analysis, normalized to β-actin levels and its ratio to that of WT littermates was displayed (N = 4–5 each, all males). Data are expressed as mean±S.E.M. (% of WT). *P<0.05, **P<0.01, ***P<0.001, compared to WT littermates by unpaired two-tailed t-test.

Recently we found that transient exposure of type-1 NRG1 protein to mouse pups produces persistent hyperdopaminergic states in the frontal cortex [27]. To assess the effects of NRG1 transgene on the dopamine system, we measured the levels of dopamine and its metabolites [dihydroxyphenylacetic acid (DOPAC) and homovanillic acid (HVA)] in various brain regions of adult mice. There were significant decreases in dopamine and DOPAC levels in the hippocampus of NRG1-Tg compared to WT mice (dopamine: *P*<0.01; DOPAC: *P*<0.001) although there were no differences in the HVA (*P* = 0.12) (**Fig. 7C**). In the frontal cortex, there was a trend toward decreasing dopamine content of NRG1-Tg mice, but not statistically significant (dopamine: *P* = 0.065; DOPAC: *P* = 0.19; HVA: *P* = 0.41) (**Fig. 7A**). In the striatum, there were no differences in the dopamine and its metabolites (dopamine: P = 0.20; DOPAC: P = 0.54; HVA: *P* = 0.88) (**Fig. 7B**).

To explore the molecular mechanism underlying the changes in dopaminergic metabolism, we examined the protein markers related to dopamine synthesis and transmission [tyrosine hydroxylase (TH), dopamine beta hydroxylase (DBH), dopamine transporter (DAT), D1 dopamine receptor (D1DR), D2 dopamine receptor (D2DR), and vesicular monoamine transporter (vMAT2)] in the frontal cortex, hippocampus and striatum. In agreement with the above change in dopamine metabolism, we found a significant protein decrease in TH, a rate-limiting enzyme of dopamine and noradrenaline synthesis, in the hippocampus (*P*<0.05) (**Fig. 7F**). The decrease in TH levels was manifested in the frontal cortex as well. In addition, we found significant increase in D2DR protein levels in the frontal cortex (**Fig. 7D**). To assess the influence on noradrenergic terminals, we also determined protein levels of dopamine-β-hydroxylase (DBH), the enzyme that converts dopamine to noradrenaline. There was no significant change in DBH levels in the frontal cortex and hippocampus (**Fig. 7D, F**). We also failed to detect significant alteration in DAT, D1DR, vMAT2 in all the regions examined (**Fig.7D–F**). These results suggest that the life-long increase in NRG1-expression disrupts dopaminergic synthesis and transmission in the cortico-limbic system. The present phenomenon contrasts our recent finding that neonatal treatment with NRG1 protein enhances dopamine synthesis and release in adulthood [27].

## Discussion

To investigate the neurobehavioral consequences of life-long NRG1 hyper-signaling, we established Tg mouse lines carrying the GFP-tagged-NRG1 (type-1) cDNA driven by the ubiquitous transcription promoter. We selected two Tg lines and backcrossed those lines with C57BL/6N mice (more than seven times) to stabilize the transgene in a single genomic integration site. One of the lines carried more copies of the NRG1 transgene than the other. In agreement, the expression of the transgene was higher in the Tg5 line compared to the Tg7 line as shown by immunoblotting as well as by real time RT-PCR. Our results indicated that the expression of the NRG1 transgene was widespread throughout the brain. As indicated the neurotrophic actions of this neurotrophic factor, the Tg mice exhibited the increase in the phenotypic markers of GABAergic neurons and oligodendrocytes [14], [15], [23], [24], [28]. In spite of the reported neurotrophic activity of NRG1 on midbrain dopaminergic neurons [29], [30], the hippocampal decrease in TH and dopamine was observed beyond our expectation.

These two independent Tg mouse lines displayed similar levels of neurobehavioral abnormalities; hyper-locomotor activity in a novel environment, learning deficits in context-fear conditioning, reduced social interactions, and a nonsignificant trend toward decreasing prepulse inhibition. Both lines also exhibited the normal behavioral phenotypes that were indistinguishable from WT littermates in acoustic startle amplitudes, vertical movement, shock sensitivity, and tone-dependent fear learning. These behavioral traits of the Tg mice appear to indicate their normal motor function or sensory abilities in a limited degree. We do not exclude the possibility that unexamined physical functions, such as olfaction, might be altered by NRG1 overexpression and influence social interaction scores of the Tg mice.

The behavioral homology between these two independent Tg lines presumably rules out the possibility that genomic disturbance of the transgene integration was involved in these behavioral deficits. In addition, it is also unlikely that the distinct genome background impurities of the two independent Tg lines resulted in the same behavioral traits. In this context, the discordant behavioral trait (social aggression) between the two Tg lines might be illustrated by the distinct genomic disturbance of the transgene integration or different background impurities of the original DBA mouse genome.

The Tg mice in the present study displayed both an increase in horizontal locomotor activity and a decrease in social behavior. Interestingly, hyperlocomotion is typically associated with positive symptoms in a mouse model of schizophrenia whereas reduced social activity is implicated as a negative symptom of this illness [31]–[33]. The hypo-dopaminergic state is often associated with impairments in social and learning behaviors, and might illustrate some of the behavioral traits of the present transgenic mice [34], [35]. In particular, their hypo-dopaminergic state in the limbic system might impair the hippocampal functions, leading to their context learning deficits [35]. The observed behavioral traits are reported in studies of various NRG1 knockout lines as well [5], [8]–[11]. Although we failed to detect significant and marginal gender x genotype interactions in individual behavioral tests, the physical examination test detected a significant main effect of gender and an interaction between Tg line and gender, presumably suggesting the dose-dependent NRG1 effects on gender-specific behavioral trends. This agrees with the reports that the down-regulation of NRG1-ErbB signaling in mice exhibit sexually dimorphic changes in several behavioral paradigms such as exploratory and habituation profiles [8], [36], [37]. The biological mechanism underlying the interaction between sex hormone and NRG1 signaling remains to be studied.

Unexpectedly, the present study and previous reports indicate that both hypomorphic and hypermorphic expression of the NRG1 gene may produce several common behavioral phenotypes in mice. This finding is quite surprising but raises a challenging question about the molecular and cellular mechanisms underlying the behavioral deficits common to both the hypermorphic and hypomorphic expression of NRG1.

PPI is also implicated in the neuropathology of schizophrenia and its animal models. In contrast to the abnormality in social behavior or locomotor activity, the PPI deficits of these Tg mice appear to be moderate. The NRG1 knockout line (transmembrane-domain of NRG1^+/−^) similarly displayed moderate or non-significant abnormality in PPI levels [2], [13]. Since there are variations in the magnitude of PPI deficits depending upon the targeted exon of NRG1 gene [4], the behavioral phenotype of the present NRG1-Tg mice is not discordant with that of NRG1 knockout mice in this context. The recent report is noteworthy that the specific overexpression of type-1 NRG1 driven by a Thy-1 promoter in brain projection neurons markedly impairs PPI [38]. The use of distinct gene promoters of EF1α and Thy-1 genes differentially regulates timing and cell types of the transgene expression and presumably results in the difference in mouse behavior. Controversy of the behavioral difference between these NRG1 Tg lines awaits further investigations, however.

NRG1 is one of the neurotrophic factors that positively regulate neuronal migration, synaptogenesis, GABAergic and dopaminergic neuronal development, and myelination [14], [15], [27]–[30], [39]–[41]. In agreement with the given biological activities of NRG1, we found the increases in parvalbumin, MBP and CNPase. These molecular phenotypes of the NRG1-Tg mice well contrast those of NRG1 knockout mouse lines. ErbB4, the receptor for NRG1 knockout-mutants exhibit reduced parvalbumin positive cells in the hippocampus [28] and loss of ErbB4 signaling by its dominant-negative form reduced oligodendrocyte number and myelination [41]. These phenotypic abnormalities of NRG1 knockout mice are in agreement with the neuropathological findings on postmortem brains of schizophrenia patients [42]–[44]. Conversely, the present NRG1-Tg mice, which display the increases in these pathological markers, may be irrelevant to an animal model for schizophrenia in spite of their schizophrenia-like behavioral deficits. In this context, it is a challenging question how the hyper-NRG1 signals reported in patients' postmortem brains is associated with the above neuropathologic deficits.

NRG1 is reported to promote cell survival of midbrain dopaminergic neurons and trigger dopamine release [29], [30], [45]–[47]. Accordingly, we had expected positive influences of the NRG1 transgene expression on the dopaminergic system in the present experiment. However, the direction of the dopaminergic changes in NRG1-Tg mice was opposite to our expectation. NRG1-Tg mice rather displayed reduction in TH protein levels and dopamine content in the hippocampus and/or frontal cortex. It is a challenging question how the hyper-signaling of NRG1 produced the TH decrease. This discrepancy might be illustrated by the NRG1 action on dopaminergic neurons [46]. NRG1 evokes an almost immediate overflow of striatal dopamine when injected into a region just dorsal to the substantia nigra. Therefore, it is possible that the life-long hyper signals of NRG1 might result in constant dopamine over-flow and produce cytotoxic influences on dopaminergic terminals [48], [49]. As there are many alternative explanations for this controversy, the exact mechanism underlying this phenomenon remains to be explored.

In summary, our behavioral results from NRG1-Tg mice and previous findings on NRG1 knockout mice highlight the complex dose dependency of NRG1 functioning in brain development or behavioral regulation.

## Materials and Methods

### Ethics statement

All of the animal experiments described were approved by the Animal Use and Care Committee guidelines of Niigata University and performed in accordance with the guidelines of NIH (USA).

### Generation of NRG1-Tg mice

The GFP gene was inserted into the NspV-SacI site between the immunoglobulin (Ig)-like and epidermal growth factor (EGF)-like domains of NRG1β1 cDNA [50]. The 2.6 kb cDNA fragment encoding mouse NRG1β1 and GFP tag was then excised by EcoRI-XbaI digestion, subcloned into the EcoRI-XbaI site of a mammalian vector pT113 (gifted from Dr. Shigekazu Nagata, Osaka University) and ligated to an EF-1α gene promoter. The DNA construct of the transgene was confirmed by DNA sequencing (data not shown). Transgenic mice were generated by pronuclear injection of the fragment (shown in **Fig. 1A**) into fertilized mouse eggs (DBA/2×C57BL/6 F1). The lines of the NRG1-Tg mice (Tg5 and Tg7, chosen for its low copy number of the transgene) were backcrossed with C57BL6NCr mice (purchased from Nihon Charles River, Yokohama, Japan) for 7–9 generations, and their offspring of heterozygous mice was used in this study. Mice were genotyped by PCR using primers corresponding to the Ig-like domain of NRG1 (forward: 5′-TGCCTCCCAGATTGAAAGAG) and the EGF domain of NRG1 (reverse: 5′-TTCTCCTTCTCCGCACACTT), giving a product with 1112 bp. All Tg mice were bred and housed under a 12 h light-dark cycle with free access to food and water. The mice were subjected to behavioral testing during the light phase between postnatal day (PND) 56–84.

### Immunoblotting

Whole brain tissues were homogenized in lysis buffer (62.5 mM Tris-HCl pH 6.8, 2% SDS, 0.5% NP-40, 5 mM EDTA) with a protease inhibitor cocktail (Roche, Indianapolis, IN, USA). After centrifugation, the supernatant was collected and protein concentrations were determined. Equal amounts of protein (30 µg/lane) were subjected to sodium dodecyl sulfate–polyacrylamide gel electrophoresis and transferred to nitrocellulose membranes. The membranes were incubated with anti-extracellular-NRG1 (7D5, 1∶1000, NeoMarkers, Fremont, CA, USA) or anti-GFP (1∶2000, Clontech, Palo Alto, CA, USA) monoclonal antibodies. Alternatively, immunoblots were probed with anti-GAD 65/67 (1∶5000, Sigma-Aldrich, St Louis, MO, USA), anti-parvalbumin (1∶10000, Abcam, Cambridge, UK), anti-MBP (1∶1000, Millipore, Bedford, MA, USA), anti-CNPase (1∶1000, Millipore), anti-NR1 (1∶250, Millipore), anti-NR2A/B (1∶150, Millipore), and anti-β-actin (1∶4000, Millipore) antibodies. Immunoblots were alternatively probed with antibodies directed against the following dopamine-related molecules; TH (1∶1000, Millipore), DBH (1∶1000, Millipore), DAT (1∶1000, Millipore), D1DR (1∶500, Santa Cruz Biotechnology, Santa Cruz, CA, USA), D2DR (1∶250, Millipore), and vMAT2 (1∶1000, Millipore). Immunoreactivity was detected by peroxidase-conjugated anti-rabbit or peroxidase-conjugated anti-mouse Ig antibody followed by a chemiluminescence reaction combined with X-ray film exposure (ECL kit; GE Healthcare, Little Chalfont, UK).

### Analysis of NRG1 mRNA expression

Real-time RT- PCR was performed in a fluorescent temperature cycler (LightCycler, Roche Molecular Biochemicals, Mannheim, Germany) according to the manufacturer's instruction. Total RNA was isolated from whole brain tissue with the guanidinium-phenol solution (Isogen, Nippon Gene, Osaka, Japan) and treated with DNase I (20 U/ml) to remove contaminating genomic DNA. NRG1 mRNA was detected by recombinant *Thermus thermophilus* DNA polymerase (High-Plus, Toyobo, Osaka, Japan) using the forward primer (5′-GCAAAGAAGGCAGAGGCAAG) and the reverse primer (5′-GCTACGGTTCAGCTCATTCC), which correspond to exon 2 and exon 3 sequences of mouse NRG1 genome, respectively. The primer set was designed to amplify mRNA transcripts specific for type-1 NRG1. RT-PCR of glyceraldehyde-3-phosphate dehydrogenase (GAPDH) mRNA was similarly carried out with the forward primner (5′-TGCACCACCAACTGCTTAGC) and the reverse primer (5′-GATGCAGGGATGATGTTCTG). These primer sets was designed to span intron(s) to distinguish PCR products of mRNA from those of genomic DNA. The lengths of the expected products were 230 bp for NRG1β1 mRNA and 239 bp for GAPDH mRNA. The genome copy number of the Tg mice was estimated by the comparative Ct method using the amplification curve of the wild genome as a standard [51].

### Physical examinations

We employed the primary behavioral screen SHIRPA developed by Rogers et al. [52] and estimate a behavioral and functional profile of NRG1 Tg mice by observational assessment. Parameters of undisturbed animals and animals submitted to battery of reflex tests are scored for quantitative analysis. The behavioral parameters assessed include posture, activity, gait, motor coordination, tremor, startle response, excitability and defecation as observed in a viewing jar and open field. Salivation, lacrimation, piloerection, placing and righting reflexes, muscle tone and other reflexes were scored by picking the animal up and eliciting the reflexes with specific equipment and manipulations [53], [54]. Naïve mice (i.e., mice not exposed to any other behavioral test) were used for these physical examinations.

### Analysis of Locomotor Activity

Exploratory motor activity was measured in a novel environment under dim light. Mice were placed in an automated activity apparatus (27 cm L×27 cm W×20 cm H, MED Associates, St. Albans, VT, USA) equipped with infrared photosensors at 1.62 cm intervals, and we measured horizontal activity every 5 min for the first hour [27]. Horizontal activity was assessed via beam crossings, which were counted by a fully automated tracking system (Activity Monitor, Med Associates).

### Measurement of acoustic startle response and prepulse inhibition

Mice were placed in a plastic cylinder and fixed in an automated startle chamber (SR-Lab Systems, San Diego, CA, USA) [27]. After a 5-min acclimation period with 70-dB-background noise (white noise), an 75-, 80-, 85-, 90-, 100-, 110-, or 120-dB white noise stimulus (40 msec duration) was given 8 times to each mouse in the same pseudo-random order at 15 sec intervals. Analysis for startle amplitudes was based on the mean of the seven trials (ignoring the first trial) for each trial type. PPI responses were measured with 120 dB acoustic stimuli combined with four different prepulse intensities. Each mouse was placed in the startle chamber (SR-Lab) and initially acclimatized for 5 min with background noise alone (70 dB white noise). The mouse was then subjected to 48 startle trials, each trial consisting of one of six conditions: (i) a 40 msec 120 dB noise burst presented alone, (ii–v) a 40 msec 120 dB noise burst following prepulses by 100 msec (20 msec noise burst) that were 3-, 6-, 9-, or 12-dB above background noise (i.e., 73-, 76-, 79-, or 82-prepulse, respectively), or (vi) no stimulus (background noise alone), which was used to measure baseline movement in the chamber. These six trial types (i–vi) were each repeated 8 times in a pseudorandom order to give 48 trials. The inter-trial interval was 15 sec. Each trial type was presented once within a block of six trials and the order of 48 trial presentations was fixed for all mice. Analysis was based on the mean of the seven trials for each trial type. The percentage PPI of a startle response was calculated as: 100 − [(startle response on prepulse-pulse stimulus trials − no stimulus trials)/(pulse-alone trials − no stimulus trials)] ×100.

### Context- and tone-dependent fear learning

The test paradigm for contextual fear conditioning was modified from procedures published in Frankland et al, 2004 [55]. Mice were placed in a shock chamber with a grid floor (10 cm L×10 cm W×10 cm H; Obaraika Ltd. Tokyo, Japan), and their baseline movement/freezing behavior was monitored for 2 min. The mice were then exposed to three rounds of 0.8 mA electric shocks (2 sec duration) with 180 sec tone cues (60 dB, 10 kHz). One day after conditioning, mice were returned to the chamber. The time spent freezing (i.e., no movement other than respiration) was recorded and scored at 30 sec intervals for 3 min. After 3 h, the mice were moved to a different chamber with a flat floor (10 cm L×10 cm W×10 cm H). In this chamber, the time spent freezing was recorded and scored for 3 min before and after the tone cue. Freezing behavior was monitored by a video camera during all sessions and analyzed by imaging software (Obaraika Ltd.).

### Isolation-induced resident-intruder test

The isolation-induced resident-intruder test to estimate social behaviors was modeled after the procedure described by Mohn et al, 1999 [56]. For one week before testing, male wild-type (WT) and NRG1-Tg mice were housed individually (resident) or in groups (intruder) of three or four mice. We note that the bedding was changed in all cages one day prior to testing. On the test day, an intruder was placed in the home cage of resident mice, and their behavior was video-recorded for 10 min. The duration and frequency of 1) anogenital sniffing, 2) sniffing of any part of resident mice excluding anogenital area (non-anogenital sniffing) and 3) non-agonistic social behaviors (grooming and lying down next to each other of resident mice) were scored by observers blind to the experimental conditions. In addition, aggressive behaviors; 1) aggressive following (resident mice rapidly follow intruder mice from behind and force it to retreat, fiercely tugging hair or tail), and 2) attacks (biting and pinning), and 3) threats (upright posture and tail rattling) were scored. Scores for each behavior were then averaged for each genotype. The experimental groups included 10 WT residents and 11 NRG1-Tg residents in the Tg5 line experiment, and 7 WT and 7 NRG1-Tg residents in the Tg7 line experiment. We purchased and used novel adult male C57BL/6 NCr mice (same age) as unfamiliar intruders.

### Quantification of dopamine and its metabolites

We measured the tissue contents of dopamine, DOPAC and HVA as described previously [57]. The prefrontal cortex, hippocampus, and striatum were dissected and frozen on dry ice. The tissue was homogenized in monoamine extraction buffer [0.1 M perchloric acid, 0.1 mM EDTA, 50 nM isoproterenol (internal standard)], incubated on ice for 30 min, and then centrifuged at 10,000×g for 10 min. Precipitates were homogenized in 0.5 N NaOH for protein determination.

The high performance liquid chromatography (HPLC) system consisted of a pump (model LC-10ADVP; Shimadzu, Kyoto, Japan), an automatic sample injector (model SIL-10ADVP; Shimadzu), and an electrochemical detector (ECD) with a glassy carbon-working electrode (model ECD-300; Eicom, Kyoto, Japan). Tissue contents of dopamine, DOPAC and HVA were measured using a C18 column (model CA-5ODS, 4.6×150 mm; Eicom). The mobile phase consisted of 50 mM trisodium citrate, 25 mM NaH_2_PO_4_, 0.03 mM EDTA, 10 mM diethylamine, 3 mM octanesulfonic acid sodium salt, 6% methanol, and 1% dimethylacetamide, pH 3.2.

### Statistical analysis

Health and physical conditions (39 parameters) were analyzed using a multiple analysis of variance (MANOVA) with genotype (two levels), line (two levels) and gender (two levels). Behavioral scores were initially analyzed using a three-way analysis of variance (ANOVA) with genotype (two levels) and gender (two levels) as the between-subjects factors and time or prepulse (four levels) as the within-subjects factors. Because the initial ANOVAs did not yield any significant results with gender, the variable was collapsed and the analysis rerun. Univariate data for the social behavioral scores, protein expression levels and monoamine contents were analyzed using an unpaired two-tailed *t* test. For post hoc testing, Fisher's LSD was used to detect differences in the absolute behavioral values. A *P-*value of less than 0.05 was regarded as statistically significant, and “N” values represent the number of animal used in the analysis. These statistical analyses were performed using SPSS 11.0 for Windows.

## Supporting Information

Table S1 Statistical values and results of MANOVA in SHIRPA test. N, animal number; AVE, average; SD, standard deviation.
(0.03 MB DOC)

Table S2 Physical and health conditions of NRG1-Tg mice in SHIRPA test. N, animal number; AVE, average; SD, standard deviation.
(0.05 MB DOC)
