# Supplementary material for: Phenotypic Characterization of Transgenic Mice Overexpressing Neuregulin-1
Source: PLoS One. 2010 Dec 9;5(12):e14185. doi: 10.1371/journal.pone.0014185 (PMC3000321; doi:10.1371/journal.pone.0014185)
Supplement: Table S1 — Statistical values and results of MANOVA in SHIRPA test. N, animal number; AVE, average; SD, standard deviation. (0.03 MB DOC) [file pone.0014185.s001.doc]

**Table S1.** Statistical values and results of MANOVA in SHIRPA test
